# Identification of MAMP-Responsive Plasma Membrane-Associated Proteins in *Arabidopsis thaliana* Following Challenge with Different LPS Chemotypes from *Xanthomonas campestris*

**DOI:** 10.3390/pathogens9100787

**Published:** 2020-09-25

**Authors:** Raeesa H. Hussan, Ian A. Dubery, Lizelle A. Piater

**Affiliations:** Department of Biochemistry, University of Johannesburg, Auckland Park 2006, South Africa; raeesahussan@gmail.com (R.H.H.); idubery@uj.ac.za (I.A.D.)

**Keywords:** affinity chromatography, innate immunity, lipopolysaccharide (LPS), pattern recognition receptors (PRRs), plasma membrane (PM), proteomics

## Abstract

Lipopolysaccharides (LPS) are recognized as microbe-associated molecular patterns (MAMPs) responsible for eliciting defense-related responses and while the effects have been well-documented in mammals, there is a lack of knowledge regarding the mechanism of perception in plant systems and recognized structural moieties within the macromolecular lipoglycan structure. Thus, identification of the LPS plasma membrane (PM) receptor(s)/receptor complex in *Arabidopsis thaliana* through proteomics will contribute to a deeper understanding of induced defense responses. As such, structurally characterized LPS chemotypes from *Xanthomonas campestris* pv. *campestris* (*Xcc*) wild-type 8004 (prototypical smooth-type LPS) and mutant 8530 (truncated core with no O–chain) strains were utilized to pre-treat *A. thaliana* plants. The associated proteomic response/changes within the PM were compared over a 24 h period using mass spectrometry-based methodologies following three variants of LPS-immobilized affinity chromatography. This resulted in the identification of proteins from several functional categories, but importantly, those involved in perception and defense. The distinct structural features between wild-type and mutant LPS are likely responsible for the differential changes to the proteome profiles, and many of the significant proteins were identified in response to the wild-type *Xcc* LPS where it is suggested that the core oligosaccharide and O-chain participate in recognition by receptor-like kinases (RLKs) in a multiprotein complex and, notably, varied from that of the mutant chemotype.

## 1. Introduction

Pathogenic microbes affect a plant’s development, reproduction, and ultimately, production yield [1] and as such, the control thereof remains a major challenge in agriculture. One such pathogen is the Gram-negative *Xanthomonas campestris* pv. *campestris* (*Xcc*), which is known to cause black rot disease amongst cruciferous plants [2]. Structurally conserved molecular signatures known as microbe-associated molecular patterns (MAMPs) exist on bacterial surfaces and in the case of Gram-negative bacteria, lipopolysaccharides (LPS) are found in the outer membrane [3]. “Smooth” type LPS comprises of amphiphilic macromolecules is composed of (i) an O-specific polysaccharide or O-chain (absent in the “rough” type LPS known as lipooligosaccharides (LOS)) (ii) a hydrophilic heteropolysaccharide, which consists of the core oligosaccharide divided into an outer and inner core, covalently linked to (iii) a lipophilic moiety termed lipid A, which acts to anchor the macromolecular LPS to the outer membrane [4,5]. LPS plays an important role in cell viability, host attachment, and bacterial virulence and activates an immune response in both mammals and plants [3]. To date, the recognition and LPS-induced signaling in mammalian cells have been well documented and involves interaction of the lipid A moiety, which forms a complex with the LPS-binding protein (LBP), CD14 receptor, Toll-like receptor 4 (TLR4)-myeloid differentiation protein 2 (MD2), and caspase-4/11. Further recognition proteins exist in mammals such as the angiogenesis inhibitor 1 (BAI1) and the cystic fibrosis transmembrane conductance regulator (CFTR) [6,7].

In comparison, the exact mechanism of LPS perception by putative receptor(s)/receptor complexes remains unknown in plants while further downstream, signaling cascades are only partially described. Hence, further studies are needed on LPS-binding/interacting proteins, which could lead to an increased understanding of plant surveillance and perception. Previous studies suggest that the plant innate immune system similarly recognizes LPS as a MAMP and known elicitor of MAMP-triggered immunity (MTI) associated with the activation of defense-related responses and expression of pathogenesis-related (PR) genes [8,9]. In this regard, LPS has been shown to be perceived by *Arabidopsis thaliana* and to elicit plant immune responses [7,8,10,11]. This LPS immune elicitation in plants is signified by a rapid influx of calcium ions into the cytoplasm as well as the production of reactive oxygen and nitrogen species (RO/NS) [7,10]. Meyer et al. [12] showed that LPS from *Xcc* induces an oxidative burst in tobacco cells, while that isolated from *Burkholderia cepacia* was found to trigger a rapid influx of calcium ions into the cytoplasm [7,10]. Recently, Iizasa et al. [13] reported that the *A. thaliana* genome contains two genes which both encode proteins of the LPS binding protein family and resemble those well-documented in mammals; namely, the LBP and the bactericidal/permeability-increasing protein (BPI). The identified genes were named AtLBP/BPI related-1 (*AtLBR-1*) and AtLBP/BPI related-2 (*AtLBR-2*).

Unlike in mammalian counterparts, the perception mechanism of different LPS moieties by plants remains debatable. Evidence suggests that the lipid A moiety as well as the intact MAMP is effective in inducing defense. In addition, synthetic oligorhamnans, similar to components of certain O-polysaccharide chains in LPS, triggered defense responses in *A. thaliana* [14,15]. Also, *B. cepacia* lipid A and O-polysaccharide moieties of LPS were reported to trigger an up-regulation of sub-sets of defense genes compared to that of the intact LPS [14]. Silipo et al. [15] showed that the intact LOS, lipid A, and core oligosaccharides of a wild-type strain from *Xcc* (8004) induce defense-related *PR1* and *PR2* genes in the leaves of *A. thaliana* in two temporal states/phases. Reportedly, the core oligosaccharide triggered gene induction in the early phases, whilst the lipid A has shown induction only in the later phases. A mutant strain, *Xcc* (8530), derived from the wildtype, is defective in the completion of its LOS, having a truncated core oligosaccharide with no O-chain and chemical modifications (degree of acylation and phosphoethanolamine substitution) in the lipid A region. The latter demonstrated to be inactive in elicitation of the *PR1* genes [15].

The current models of immunity postulate that MAMP perception in plants occurs through pattern recognition receptors (PRRs), which comprises of the receptor-like kinases (RLKs) and receptor-like proteins (RLPs) found in, or associated with, the plasma membrane (PM) [5,16]. Sanabria et al. [11] demonstrated the role of an S-domain RLK (SRK) in *Nicotiana tabacum* when induced with LPS from *B. cepacia*, with up-regulation of the *Nt-Sd-RLK* (*N. tabacum* S-domain-receptor-like kinase). Desaki et al. [3] demonstrated that the carbohydrate/peptidoglycan (CO/PGN) co-receptor OsCERK1 plays a role as either receptor or co-receptor in LPS perception in rice but differs from that in Arabidopsis. In the latter, the RLK, LORE (LIPOOLIGOSACCHARIDE-SPECIFIC REDUCED ELICITATION), which belongs to a specific class of plant bulb-type (B-type) lectin S-domain (SD)-1 kinases, was reported to detect the LPS of *Pseudomonas* species and *X. campestris*, thereby triggering responses in the form of MTI [17]. This LORE-response was, however, recently shown to rather be triggered from co-purified medium-chain 3-hydroxy fatty acid (mc-3-OH-FA) metabolites [18] and thus, LORE is not the LPS receptor in *A. thaliana*.

Previously in our group, a novel affinity chromatography strategy [9] successfully captured proteins such as the BRASSINOSTEROID INSENSITIVE 1-associated receptor kinase 1 (BAK1; At4g33430) involved in LPS signaling (resembling flg22-based flagellin sensing), while Baloyi et al. [19] identified BAK1 as well as a lectin SRK in *A. thaliana* plants treated with LPS from *Escherichia coli*. In this study, a similar affinity chromatography approach was followed in order to expand on the functional role of the LPS moieties since it remains elusive which PRR recognizes/binds to the MAMP moiety(ies). The study thus aimed to capture/enrich and identify LPS-interacting proteins from the *A. thaliana* plasma membrane (PM) and associated fractions following treatment with LPS chemotypes (*Xcc* 8004 and *Xcc* 8530) in order to investigate the proteomic environment surrounding a potential receptor/receptor complex possibly involved in the concomitant perception(MAMPs).

## 2. Results

### 2.1. Isolation and Verification of the Plasma Membrane (PM)-Associated Fraction from A. thaliana Following LPS Treatment

The PM is the interface of communication between the plant cell and its surrounding environment [20]. The plant PRRs (RLKs and RLPs) involved in immune and defense responses have been identified either on or associated with the PM [5]. The findings from Vilakazi et al. [9] on the LPS*_B. cepacia_*-interacting proteins in *A. thaliana* suggests that the perception of the lipoglycan could possibly occur within membrane rafts/microdomains. Furthermore, the authors identified BAK1 as an interacting protein as supported by Baloyi et al. [19]. In the latter study, PM proteins identified from *A. thaliana* leaves following LPS*_E. coli_* treatment also indicated that the perception and resulting signal transduction occurs via PM proteins most likely within the specialized raft perception domains. Thus, the PM-associated fraction is the focus of this study since it is speculated that the LPS receptor/receptor complex may be localized within. A small-scale sucrose-density gradient centrifugation procedure for the enrichment and isolation of the PM-associated fraction from *A. thaliana* leaf tissue was used as an alternative to the aqueous two-phase partitioning due to the latter requiring large amounts of starting material [21]. Giannini et al. [22] has previously shown this method to be as effective and reproducible as the aqueous two-phase partitioning method in isolating and enriching the PM. Successful enrichment was supported (Figure S1) by the reduction in the number of protein bands and relative intensity thereof across the various fractions (HM, MF, and PM). In addition, confirmation of the successful isolation of enriched fractions was routinely verified by MAPK Western blot analyses for little to no presence of MAPK in the MF and PM (Figure S2). For the purpose of this study, it is important to note that a PM-associated fraction is of more interest than a pure PM fraction. The goal was not to achieve absolutely pure PM fractions, but also to include proteins that are loosely associated with the PM that may play important roles in membrane-specific recognition sites and thus, part of an associated-receptor complex for LPS perception.

### 2.2. Identification of the PM-Associated LPS-Interacting Candidate Proteins of A. thaliana Following Enrichment by Affinity Chromatography

The affinity chromatography strategies were employed as a means of capturing LPS-interacting PM-associated candidate proteins for both LPS chemotypes. Control matrices, with no immobilized LPS, were included in parallel for all affinity capture steps. In the three different affinity strategies, various moieties of the LPS chemotypes were immobilized, thus resulting in various structural features to serve as bait (as elaborated on in the Discussion and Methodology sections), in order to capture interacting PM-associated proteins. In addition, these three dissimilar affinity matrices were employed in order to increase the likelihood of identification of the LPS-interacting PM-associated candidate proteins.

#### 2.2.1. Detoxi-Gel™ Endotoxin Removing Gel Affinity Method

The Detoxi-Gel™ Endotoxin gel targets the lipid A domain of LPS, leaving the core and O-polysaccharide free for interaction. A representation of the protein elution profiles due to the binding and elution events at the 6 h time point of the 0–24 h study is illustrated in the representative supplementary Figure S3A,B, along with the elution profile for the control (Figure S3E: no immobilized LPS).

Tables S1 and S2 in the supplementary data file list the identified candidate LPS-interacting PM-associated proteins from LC/MS/MS (subsequent to affinity enrichment following treatment of *A. thaliana* with LPS chemotypes from *Xcc* 8004 and *Xcc* 8530, respectively), after taking the Byonic™ scores and log probability thresholds into consideration. These were compared to the control (non-specific binding to polymyxin resin with no LPS immobilization) proteins in Table S3, with the latter thus not considered as interacting candidate proteins. In addition, the proteins identified outside the threshold, i.e., proteins with low Byonic™ scores, were tabulated but not reported.

#### 2.2.2. The EndoTrap^®^ HD Endotoxin Removal Affinity Chromatography

For this strategy, leaving the lipid A and O-chain polysaccharide as baits, the LPS chemotypes (*Xcc* 8004 and *Xcc* 8530) were first complexed with the PM-associated protein fraction to allow interaction prior to enrichment of the candidate LPS-interacting proteins. The elution profiles obtained from the spectrophotometric analyses of the binding and elution events of a representative 6 h time study are illustrated in supplementary Figure S4A,B. In addition, the elution profile for the control, which was used as a measure of non-specific binding of PM proteins to the EndoTrap^®^ bacteriophage-derived protein resin (no LPS immobilization), is illustrated in Figure S4C.

The PM-associated candidate LPS-interacting proteins identified (subsequent to interaction with the EndoTrap^®^ affinity chromatography system following treatment with *Xcc* 8004 and *Xcc* 8530 LPS chemotypes, respectively) are tabulated in Tables S4 and S5. The results show that more proteins were captured and identified from the PM fractions with the LPS chemotype *Xcc* 8004 ligand than for the LPS chemotype *Xcc* 8530 counterpart. This can be attributed to the significant structural differences in the LPS bait-moieties of the wildtype vs. mutant. The identified proteins with low scores were tabulated but not reported, while those from the control (non-specific binding to resin with no LPS immobilization) are listed in Table S6 and were not considered as interacting candidate proteins.

#### 2.2.3. The MagReSyn™ Streptavidin Magnetic Polymeric Microsphere Affinity Chromatography

In this strategy, the LPS chemotypes from *Xcc* 8004 and *Xcc* 8530 were biotinylated by a transesterification reaction prior to immobilization. Here, the lipid A and O-chain polysaccharide chains are biotinylated, thus allowing the capture of candidate LPS–interacting PM proteins through the core oligosaccharide of the LPS [9,23]. Representative Figure S5A,B illustrates the spectrophotometric analysis of the elution profiles obtained by the binding and elution events in response to the change in eluents for the 6 h time study. Figure S5C includes the control that was used to identify the non-specific interaction between the MagReSyn™ streptavidin polymeric microspheres (no LPS immobilization) and the PM proteins of *A. thaliana*.

Significant PM-associated LPS-interacting candidate proteins identified by LC/MS/MS (following treatment with LPS chemotypes *Xcc* 8004 and *Xcc* 8530, respectively) are tabulated in Tables S7 and S8 following consideration of Byonic^TM^ scores and log probabilities above the threshold. The low score counterpart proteins were tabulated but not reported, while those that interacted with the MagReSyn streptavidin microspheres (no LPS immobilization) are tabulated as control proteins in Table S9 and were not considered as interacting candidates.

### 2.3. Common Identified Candidate LPS-Interacting PM-Associated Proteins Following Enrichment Approaches

#### 2.3.1. Assessment of the Three Affinity Chromatography Strategies

**Figure 1:**
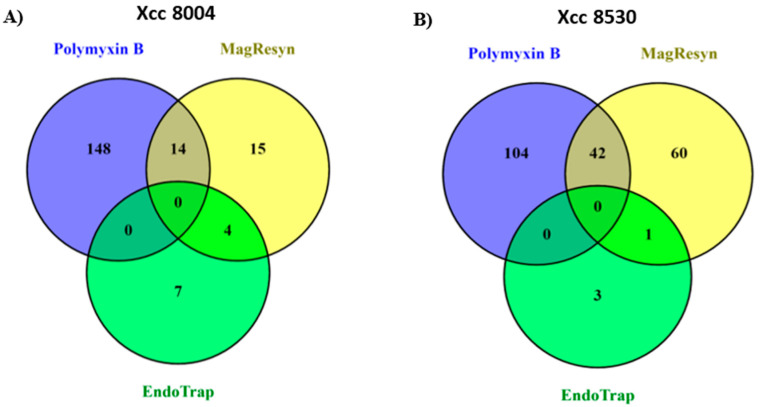
Venn diagrams showing the overlapping/common (numbers in intersection) and distinct (numbers in the circle) candidate Lipopolysaccharides (LPS)-interacting Plasma Membrane (PM)-associated proteins identified by the three enrichment strategies; blue: polymyxin B, yellow: MagReSyn™ streptavidin magnetic polymeric microsphere, and green: EndoTrap^®^ HD Endotoxin removal affinity chromatography for the (**A**) LPS chemotype *Xcc* 8004 and (**B**) LPS chemotype *Xcc* 8530.

Three enrichment strategies, complementing the different immobilized and available (bait) moieties of the two LPSs, were investigated in order to determine if similar or different PM-associated proteins will be identified when the various molecular signatures of the MAMP are immobilized to the affinity resins. Accordingly, an LPS-interacting protein that is found to be common in two or more affinity enrichment strategies could be considered more significant than that being identified in only one approach. Here, the common/shared enriched LPS-interacting PM-associated proteins in two or more affinity strategies are suggestive of proteins that participate in “complexes” when perceiving the LPS chemotypes. However, individually enriched proteins also cannot be ignored, given the possible effects of the LPS moieties on *A. thaliana* PM MAMP perception. Comparative analysis of the three affinity enrichment strategies are shown for the two *Xcc* LPS chemotypes in Figure 1, with the numbers of common candidate LPS-interacting PM-associated proteins found overlapping and listed in Table 1 and Table 2, with the supporting data in the Supplementary Data files. The family and subfamily proteins with the same function were considered and grouped as one, regardless of the different accession numbers, in order to make the comparative analysis less redundant.

#### 2.3.2. Comparison between the Two *Xcc* LPS Chemotypes

**Figure 2:**
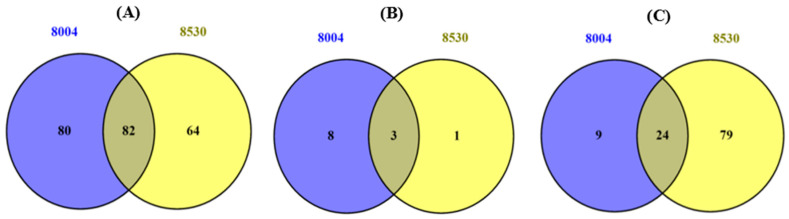
Venn diagrams showing the overlapping/common (numbers in intersection) and distinct (numbers in the circle) candidate LPS-interacting PM-associated proteins when comparing the LPS chemotypes *Xcc* 8004 (blue) and *Xcc* 8530 (yellow) for the affinity strategies (**A**) polymyxin-B immobilized, (**B**) EndoTrap^®^ HD Endotoxin removal, and (**C**) MagReSyn™ streptavidin magnetic polymeric microsphere affinity chromatography.

Comparative analysis between the two LPS chemotypes *Xcc* 8004 (wild-type) and *Xcc* 8530 (mutant) revealed common LPS-interacting PM-associated candidate *A. thaliana* proteins for all the affinity enrichment strategies as illustrated by the Venn diagrams in Figure 2. Each LPS chemotype is possibly perceived in a different manner by *A. thaliana* cells due to the different moieties present in each [15]. Based on this, the different moieties of LPS may play a role in its perception as a MAMP, however this does not rule out the possibility that common associated proteins may be present in the receptor/recognition complex at the PM. Here, the common LPS-interacting PM-associated proteins between the two LPS chemotypes in functional categories such as perception/signaling and response/defense suggest that even though certain moieties may be absent from the mutant LPS chemotype *Xcc* 8530, the same PM-associated proteins as found for the wild-type may be present. The identities of the common candidate interacting PM-associated proteins for both LPS chemotypes are tabulated in Table 3, Table 4 and Table 5.

## 3. Discussion

### 3.1. Analysis of the Candidate LPS-Interacting PM-Associated Proteins from A. thaliana within Functional Categories

The PM is the main cellular interface which mediates communication and is the primary site for perception of external signals. As such, this membrane partakes in many biochemical processes, which are primarily controlled by the PM-associated proteins [24]. These proteins have been implicated to function in membrane transport, trafficking, endocytosis, and maintaining electrochemical gradients perception and further signal transduction in which sensing and responding to biotic stresses takes place [25]. Furthermore, the plant innate immune receptors and defense response regulators are likely associated with the PM [24]. In this study, affinity chromatography enrichment strategies were specifically designed to capture candidate LPS-interacting PM-associated proteins to gain a deeper understanding of the PM proteome related to the immunity and defense responses that are associated with the LPS chemotypes *Xcc* 8004 and *Xcc* 8530, ultimately to gain more insights into the mechanism of LPS perception at the molecular level. The candidate LPS-interacting PM-associated proteins that were identified by the above-mentioned strategies and reported in Table 1, Table 2, Table 3, Table 4 and Table 5 and Tables S1, S2, S4, S5, S7 and S8 are discussed in terms of the functional categories in the following sections.

#### 3.1.1. Perception and Signaling

The first line of plant immunity involves innate immune receptors that possess the ability to recognize and detect MAMPs of pathogenic microbial invaders in the form of PM-resident PRRs. The currently known PRRs belong to either of the RLKs and RLPs, which upon perception and binding, transduce secondary signals, which results in MTI [26].

In this study, several RLKs have been identified as candidate LPS-interacting PM-associated proteins belonging to different classes *viz*., the LRR-RLK and G-type LecRLK protein kinase family members. A negative regulator of MTI, the inactive LRR RLK BIR2 (BAK1-interacting receptor-like kinase 2) and LRR-RLK SOBIR1, was identified (Table S1; polymyxin B and LPS Xcc 8004). LRR RLK BIR2 is known to regulate the complex formation by binding to the brassinosteroid-insensitive 1 (BRI1)-associated kinase BAK1 in the cells’ resting state when no ligand is available, thus ensuring that BAK1 does not bind to the PRR [16]. LRR-RLK SOBIR1, also related to BAK1-mediated signaling, is thought to negatively regulate the plant resistance signaling pathways by counteracting BIR1 (BAK1-interacting receptor-like kinase 1) to promote cell death and disease resistance. SOBIR1 has an essential role as a regulatory RLK for the stability of certain RLPs and thus partakes in RLP-mediated immunity [27]. A study by Vilakazi et al. [9] related a BAK1 signaling mechanism to LPS perception and this protein was suggested as one of the transmembrane proteins that potentially interacts with LPS from *B. cepacia* and this recognition may be analogous to that of flg22. The probable LRR-RLK, At1g53430 (Table 3; polymyxin B and both chemotypes), consists of a malectin domain able to bind to carbohydrate ligands [28], while LRR-RLK At3g14840, also identified in *A. thaliana* (Table 3 and Table S8; MagReSyn^TM^ and LPS *Xcc* 8530), has been recognized as a putative N-glycosylated integral PM protein [29]. A study by Xu et al. [30] demonstrated the role of this RLK and BR signaling kinase 3 (BSK3). It was observed that the expression levels were increased by oomycete infection, thus suggesting a role for this BSK3-interacting RLK in plant immunity.

The lectin domain (Lec)-RLKs are as important as other PRRs for adaptation to environmental cues, pathogen detection, and plant disease resistance [5,31]. Even though an Arabidopsis bulb-type lectin S-domain RLK (At1g61380/SD 1-29/LIPOOLIGOSACCHARIDE-SPECIFIC REDUCED ELICITATION, LORE) was implicated in sensing the lipid A moiety of LPSs from *Pseudomonas* and *Xanthomonas* species [17], this was recently ascribed to the presence of co-purified medium-chain 3-hydroxy fatty acids (mc-3-OH-FAs) [18]. A G-type LecRLK, At1g67520, was solely identified (Table S1; polymyxin B) following LPS *Xcc* 8004 treatment. The encoding gene has been found to be up-regulated in a study by Iizasa et al. [32], where transcriptomic changes in the *Arabidopsis atlbr-2* mutant (lacking LBP, LPS-binding protein/BPI-related-2) was observed when being treated with LPS from *P. aeruginosa*. Additional G-type LecRLKs were identified, with a S-domain RLK, namely G-type LecRLK At4g27300/ SD1-1 and G-type LecRLK At1g11330/SD1-13 (Table S1; polymyxin B and LPS *Xcc* 8004). The latter is the nearest homolog to the *N. tabacum* Nt-SD-RLK proposed by Sanabria et al. [11] to be involved in LPS perception. This study points to the role that S-domain RLKs might play in MAMP perception of LPSs and further induction of signal transduction events due to LPS perception. The study showed an early up-regulation of the *Nt-SD-RLK* gene, which encodes an S-domain RLK from *N. tabacum*. An L-type lectin domain-containing RLK from clade IV.1 was identified in *A. thaliana* (Table 3; polymyxin B and both LPS chemotypes) and another from clade VII.1 (Table S1; polymyxin B and LPS *Xcc* 8004). Experimental studies suggest that the L-type lectins have been implicated in playing a role in defense and plant-pathogen interactions, thus establishing a common role in plant immunity [31].

Calcium dependent protein kinases (CDPK) 9, 3, 15, and 21 were identified in *A. thaliana* samples (Tables S1 and S2; polymyxin B and each LPS chemotype, respectively), with 3, 15, and 21 identified as common candidate LPS-interacting PM-associated proteins (Table 3; polymyxin B and both LPS chemotypes). In *A. thaliana*, the overexpression of *AtCDPK1* was found to aid in conferring resistance to broad-spectrum bacteria and fungi [33]. This thus suggests that CDPKs are important transducers of the MAMP-induced signals to trigger MTI.

The putative LBP/BPI binding protein, At1g04970 was identified as a common candidate LPS-interacting PM-associated protein (Table 1, Table 2, Table 3, Table 5; both LPS chemotypes and all enrichments except EndoTrap^®^**)**. Iizasa et al. [15] demonstrated for the first time that the N-terminal domain of the AtLBP/BPI-related1 (AtLBR1) in *A. thaliana* binds directly to the LPS. LPS-treated AtLBR mutants were also utilized and showed a delayed expression of immune responses such as *PR1* gene expression, ROS, and NO and it was further demonstrated that AtLBRs were able to bind to both rough (no O-chain present) and smooth LPS (all moieties present). Therefore, it is plausible that the AtLBR1 was identified in PM-associated samples treated with LPS chemotypes *Xcc* 8004, in which all LPS moieties were present, as well as *Xcc* 8530, in which the O-chain was absent. This suggests a probable role of AtLBR1 in the recognition of *Xanthomonas* LPS by *A. thaliana*.

Another protein that is involved in signaling is the general regulatory factor 10 or 14-3-3 protein, which was also identified (Table S1; polymyxin B and LPS *Xcc* 8004). This protein is involved in brassinosteroid signaling by interaction with the brassinosteroid signaling positive regulator family protein, BZR1 [34].

#### 3.1.2. Defense and Stress Response

In plants, lectins play important roles in defense and are induced and expressed in plants in response to environmental stress as well as pathogen attack [31]. Carbohydrates are the potential ligands of lectins and are present in core and O-chain of glycoconjugates like LPS. Lectins may therefore be involved in LPS perception [4,31]. In this study, Jacalin-related lectins (JRL) (Table 2; polymyxin B and MagReSyn^TM^ for LPS *Xcc* 8530, Table 3; polymyxin B and LPS *Xcc* 8004 and Tables S4 and S8) and mannose binding lectins (Table 1; EndoTrap^TM^ and MagReSyn™ for LPS *Xcc* 8004) have been identified as candidate LPS-interacting PM proteins. The identified JRLs and mannose-binding lectins suggest that lectins in general may play a role in surveillance and perception of the MAMPs when *A. thaliana* was treated with both LPS chemotypes from *Xcc* 8004 and *Xcc* 8530. These two protein families may furthermore play a role in the defense-related responses when confronted with biotic stresses and recognizing pathogen attack, since both have been identified amongst the most prominent lectins.

The dehydrins were identified as candidate LPS-interacting PM-associated proteins (Tables S1 and S2; polymyxin and each LPS chemotype respectively, and Table 2; polymyxin and MagReSyn^TM^ for LPS *Xcc* 8530). Yang et al. [35] has demonstrated the role that dehydrins play in biotic stresses, where DHN1 was upregulated by the phytohormones methyljasmonate and salicylic acid, which are known to be involved in orchestrating plant defense responses.

Myrosinases were identified as candidate LPS-interacting PM proteins (Table 4; EndoTrap^®^ for both LPS chemotypes and Table S8; MagReSyn™ and LPS *Xcc* 8530). Myrosinases are involved in the plant defense system and work in conjunction with glucosinolates produced by Brassicaceae species against biotic diseases. The dual glucosinolate-myrosinase system acts against biotic defense by catalyzing the breakdown of the glucosinolates upon plant damage, thereby producing hydrolysis products, which are toxic to pathogens and herbivores, and acting as a plant defense system to certain microbial pathogens [36].

Remorin family proteins were identified (Table 2 and Table 3; polymyxin for both LPS chemotypes and Table S8; MagReSyn^TM^ for LPS *Xcc* 8530). Remorins were identified by Vilakazi et al. [9] who also used LPS from *B. cepacia* as a MAMP. It is likely that remorins were identified as PM/raft proteins by both LPS chemotypes, regardless of altered structural moieties. The plant remorin proteins have been detected in lipid rafts as detergent-insoluble membrane fractions and are thus referred to as PM markers [19] and function in microbial infection and plant signaling processes and can interact with several RLKs [37].

The hypersensitive-induced response (HIR) proteins 3 and 4 were identified as candidate LPS-interacting PM-associated proteins (Table 2, Table 3 and Table 5; polymyxin and MagReSyn^TM^, respectively, for both LPS chemotypes). Studies have shown that an HIR gene in pepper, when over-expressed, caused enhanced disease resistance to *P. syringae* pv. *tomato* (*Pto*) DC3000 and in *A. thaliana*, four HIR family genes are found, namely, *AtHIR1*, *AtHIR2*, *AtHIR3*, and *AtHIR4* [38]. AtHIR proteins are associated with the membrane-associated disease resistance protein *P. syringae* protein 2 (RPS2), which belongs to the coiled coil (CC)-NB-LRR subclass of the NB-LRR family. Similar proteins were identified by Baloyi et al. [19] and Vilakazi et al. [9] using LPS as a MAMP. In the latter citation, HIR proteins 3 and 4 were also found common to the polymyxin B immobilized affinity and MagReSyn^TM^ affinity systems [9].

Callose synthase 10 and 12 were commonly identified as candidate LPS-interacting PM-associated proteins (Table 3; polymyxin and both LPS chemotrypes). The production of callose in a plant is one of the hallmarks of plant defenses when leaves are affected by pathogens and prevents the penetration of bacteria and as such, the deposition of callose has been implicated as a defense-related response to LPS [39].

Resistance (*R*) genes encode R proteins that enable plants to recognize the presence of pathogens and activate inducible defenses [40]. The disease resistance RPP8-like proteins were identified (Tables S1 and S2; polymyxin and both LPS chemotypes). Also, noteworthy, although perception of LPSs involves PM-associated proteins, triggered immune signaling may also lead to the induction of pathogenesis-related (PR) proteins, as reported in several plant systems [39,41].

#### 3.1.3. Membrane Transport and Trafficking

A multitude of ion channels are activated in response to pathogen attack and plant defense-related responses as previously mentioned, while anion channels are activated downstream of PRRs [42,43]. Chloride channels, potassium channels, and cation channels were identified as candidate LPS-interacting PM-associated proteins (Table 3; polymyxin and both LPS chemotypes). The latter have been shown to be required for MTI [41]. Transporter proteins were identified as candidate LPS-interacting PM proteins viz., ammonium transporter-1-like proteins (Table 2 and Table 3; polymyxin and both LPS chemotypes and Table S8; MagReSyn™ and LPS *Xcc* 8530), monosaccharide transporters (Table S1; polymyxin LPS *Xcc* 8004), PM-type ATPases transporters (Table 1; MagReSyn™ and EndoTrap^®^ for LPS *Xcc* 8004 and Table 5; MagReSyn™ for both LPS chemotypes), calcium transporting ATPases (Table 1; MagReSyn™ and EndoTrap^®^ for LPS *Xcc* 8004 and Table 5; MagReSyn™ for both LPS chemotypes), and the ATP-binding cassette (ABC) transporters (Table 3, polymyxin and both LPS chemotypes and Table S8; MagReSyn™ and LPS *Xcc* 8530). The abovementioned transporter proteins are involved in the plant immune system and responses to various biotic stresses, thus contributing to antibacterial defense and plant immunity [44,45].

Aquaporins are membrane water channel proteins that not only mediate water uptake controlled by the PM intrinsic proteins (PIP), but also play important roles in plant defense against biotic stresses as dehydration occurs as a result of pathogen infection and affects plant water homeostasis and have been characterized as PM markers [46]. Aquaporins PIP 1 and 2 were identified as common candidate LPS-interacting PM proteins (Table 3; polymyxin and both LPS chemotypes, Table S4; EndoTrap^®^ and LPS *Xcc* 8004 and Table S8, MagReSyn^TM^ and LPS *Xcc* 8530), but in the EndoTrap^®^ protocol for only the 0 h LPS *Xcc* 8004 sample, while that for MagReSyn^TM^ was in the 6 h LPS *Xcc* 8530 sample.

The membrane trafficking events to and from the PM are altered when plant cells are exposed to pathogenic microbes and adjusting vesicle membrane trafficking in a plant allows cells to respond to microbial challenge and aims to limit pathogenesis [47]. The events are controlled by the following regulatory proteins; ADP-ribosylation factors (ARF1), which mediate the budding of transport vesicles but also function in plant disease resistance. This protein was found as a common candidate LPS-interacting protein (Table 5; the MagReSyn™ and both LPS chemotypes). The Rab small GTPases are involved in regulatory trafficking steps such as targeting, tethering, and docking of transport vesicles to the target membrane [48]. Rab GTPases play roles in defense-related responses as they are involved in the secretion of immunity-related proteins to the PM [49]. A number of Rab GTPase subfamilies were identified as common candidate LPS-interacting PM-associated proteins (Table 1 and Table 3; polymyxin and both LPS chemotypes and Table 5; MagReSyn™ and both LPS chemotypes). The tethering proteins such as Exocyst 70 assist in tethering the secretory vesicles to the PM. This specific protein has been shown to be involved in plant-pathogen interactions according to Inada and Ueda [48], and Gu et al. [50] states that Exo proteins are likely to carry defense cargo and contribute to secondary defense. Exocyst complex proteins (70 family protein, EXO 70A1, and complex component sec 6) were identified as candidate LPS-interacting PM-associated proteins (Tables S2 and S8). The SNAREs are responsible for the membrane fusion of transport vesicles to the target membrane [48]. V-snares or vesicle SNAREs (R-SNAREs) and T-SNAREs or transmembrane SNAREs include syntaxins [49]. These have also been implicated in mediating defense-related responses. A study by Kalde et al. [51] has demonstrated that syntaxin of plants SYP132 is involved in defense against bacterial pathogens in *N. benthamiana*. The authors further implicated that *NbSYP132* was involved in the exocytosis pathway as the target for vesicles containing antimicrobial PR proteins. Syntaxin 132 was identified as a common candidate LPS-interacting PM-associated protein (Table 2 and Table 3, and Table S8; polymyxin and MagReSyn^TM^ for both LPS chemotypes).

The endocytic trafficking pathway plays a role in immunity and microbial pathogenesis at the cytosolic surface of the PM [49,50]. Recycling and degradation are processes encompassing clathrin-mediated endocytosis, which is an important mechanism involved in plant–microbe interactions which could terminate or sustain defense-related signaling [47,48]. Clathrin light and heavy chains facilitate the clathrin-coated vesicles that participate in internalization of bound ligands. Mgcina et al. [52] suggested that when LPS binds to mesophyll protoplasts in *A. thaliana*, the MAMP binding sites are internalized by endocytosis, leading transiently to reduced levels of the said sites. Clathrin light chain (1, 2, and 3) were identified as candidate LPS-interacting PM-associated proteins (Table S2; polymyxin and LPS *Xcc* 8530).

Patellins were identified as candidate LPS-interacting proteins (Table 2 and Table S8; MagReSyn™ and LPS *Xcc* 8530 and Table 3; polymyxin and both LPS chemotypes). According to Tejos et al. [53], the patellins are involved in a host of functions, including diverse signaling pathways and pathogen attack, polarity, and patterning. Vilakazi et al. [9] also identified these proteins as LPS-interacting PM-associated proteins using similar affinity strategies following *A. thaliana* treatment with *B. cepacia* LPS.

A last noteworthy observation is that membrane transport and trafficking proteins were most-commonly identified in two (polymyxin and MagReSyn^™^) of the three affinity strategies. The EndoTrap^®^ HD affinity system resulted in the identification of only a few candidates in this category. It is tempting to speculate that this can be attributed to the mutant LPS (chemotype *Xcc* 8530) having fewer and different available moieties due to the absence of the O-chain and differences in the molecular configuration of lipid A, however initial perception compared to downstream signaling responses/effects should not be confused.

## 4. Materials and Methods

### 4.1. LPS Extraction, Characterization, and Derivatization

The LPSs from two strains of *Xanthomonas campestris* pv. *campestris*, wild-type (*Xcc* 8004), and mutant (*Xcc* 8530) were extracted and purified using the hot-water phenol method [54] and characterized for the total carbohydrate content [55] and Kdo content [56], alongside a control LPS from *Burkholderia cepacia* (data not shown). The samples (1 mg/mL) were subjected to sodium dodecyl sulfate polyacrylamide gel electrophoresis (SDS-PAGE) on 12.5% gels to further characterize the LPS. Silver-periodate staining [57] was performed in order to visualize the banding patterns of the LPS bands/moieties (Figure S6A).

The derivatization of the *Xcc* LPSs (Figure S6B) was performed according to Giangrande et al. [23] via a transesterification reaction with a biotin-*p*-nitrophenyl ester. Then, 15 mg of the lyophilized LPS were solubilized in 1 mL pyridine (Sigma Aldrich, St Louis, MO, USA), after which 45 mg of the biotin-*p*-nitrophenylester (Sigma Aldrich, St Louis, MO, USA) was added in a 1:3 (*w*/*w*) LPS/biotin-*p*-nitrophenylester ratio. The reaction was performed in the dark for 2 h at 80 °C. The sample was then dried under nitrogen and dissolved in MilliQ dH2O. Seven kDa MWCO Zeba™ spin desalting columns (Thermo Scientific, Waltham, MA, USA) were used to remove the excess reagents. Once the sample cooled to room temperature (RT), 130 µL was applied to the top of the column followed by centrifugation for 2 min at 1500× *g*. The fractions of the LPS were pooled and lyophilized.

### 4.2. Plant Growth and MAMP Treatment

*Arabidopsis thaliana* (Colombia-O) wild-type seeds were sowed into pots which contained Culterra™ germination mix soil (Culterra, Muldersdrift, South Africa). The plants were incubated in a controlled growth room of 22–23 °C under a 12 h light/12 h dark cycle, watered twice every week, and the seedlings were also fertilized two times a week or as required with a 1:300 (*v*/*v*) dilution of Nitrosol^®^ Natural Organic Plant Food (Efekto, Johannesburg, South Africa). The plants were consistently monitored for signs of contamination or infection. When the maturity stage with fully developed rosettes was reached, plants were treated with 100 µg/mL of *Xcc* LPSs from both wild-type and mutant strains by pressure infiltration into the abaxial side of the leaves (this was performed as a pre-treatment to increase the concentration of putative receptor proteins able to interact/bind LPS [11]. The MAMPs were prepared by solubilizing 100 µg/mL of LPS in 2.5 mM magnesium chloride, MgCl_2_ (Saarchem, Johannesburg, South Africa). To minimize biological variation, three biological repeats of LPS treatment (*Xcc* 8004 vs. *Xcc* 8530) were conducted for the time study (0, 6, 12, 18, and 24 h) along with the respective 2.5 mM MgCl_2_ controls containing no LPS.

### 4.3. Small Scale Isolation of the Plasma Membrane (PM)-Associated Fraction

The PM-associated fraction from *A. thaliana* leaves was isolated according to a small-scale procedure described by Giannini et al. [22] and Abas and Luschnig [58] and has been applied in similar PM investigations [9]. Approximately 20 g of treated leaves and controls for each of the respective time points were ground to a powder in liquid nitrogen using a mortar and pestle, followed by homogenization in 50 mL of homogenizing buffer [250 mM sucrose, 3 mM ethylenediaminetetraacetic acid (EDTA) (Saarchem, Johannesburg, South Africa), 10% (*v*/*v*) glycerol, 0.5% (*w*/*v*) (poly(vinylpolypyrrolidone) (PVPP) (Sigma, St. Louis, MO, USA), 2 mM phenylmethylsulfonyl fluoride (PMSF) (Boehringer Mannheim, Mannheim, Germany), 15 mM β-mercaptoethanol (Calbiochem, San Diego, CA, USA), 4 mM dithiothreitol (DTT) (ThermoFisher Scientific, Loughborough, UK), 250 mM potassium iodide, KI (Saarchem, Johannesburg, South Africa), 70 mM Tris-HCl (Merck, Modderfontein, South Africa), pH 7.5] at 4 °C using an ultraturrax homogenizer (CAT X120, Paso Robles, CA, USA). The leaf homogenate was filtered through two layers of miracloth (Millipore/Merck, Darmstadt, Germany) and resulted in a homogenate fraction (HM) which was further centrifuged at 6000× *g* for 3 min at 4 °C using a fixed angle high-speed centrifuge. The pellet was discarded, which contained the cell debris and nuclei, and the supernatant was subjected to another round of centrifugation at 13,000× *g* for 25 min at 4 °C. The supernatant was then discarded and the pellet re-suspended in 800 µL of ice cold microsomal suspension buffer [250 mM sucrose, 10% (*w*/*v*) glycerol, 1 mM DTT, 1 mM PMSF and 2 mM 2-(N-morpholino) ethanesulfonic acid (MES) (Sigma Aldrich, St Louis, MO, USA)] to obtain the microsomal fraction (MF). Following this, 500 µL of the MF was layered onto sucrose consisting of 700 µL of 38% (*w*/*v*) and 700 µL of 25% (*w*/*v*) in 1 mM Tris/MES, pH 7.2 and 1 mM EDTA solution to create a discontinuous gradient. This was then centrifuged at 13,000× *g* for 1 h at 4 °C. After centrifugation, the PM appeared as a band at the 25%/38% interface and was aspirated with a pipette. To validate the successful isolation procedure of the PM-associated fraction, MAP kinase (MAPK) Western blot analysis was performed [9,19]. A 12% SDS-PAGE gel (1-DE) coupled to densitometric analyses was performed with each of the said fractions (HM, MF, and PM) and was stained with Fairbanks Coomassie [59] as a further means of validation of the small-scale isolation method [60].

### 4.4. Affinity Chromatography

Affinity chromatography was performed by employing three affinity matrices and processes, namely: (i) EndoTrap^®^ HD Endotoxin removal (Hyglos, Deggendorf, Germany) method, (ii) Detoxi-Gel™ Endotoxin removing gel procedure (Thermo Scientific, Waltham, MA, USA), and (iii) MagReSyn™ streptavidin magnetic polymeric microspheres (Resyn Biosciences, AEC-Amersham, Midrand, South Africa) in a similar manner to Vilakzi et al. [9]. For all of the affinity approaches, the LPS from both the *Xcc* 8004 and 8530 strains were individually employed to serve as bait by being immobilized to the different affinity resins/matrices, after which both the *Xcc* 8004 and 8530-treated PM-associated fractions were passed through the affinity columns and candidate LPS-interacting proteins subsequently identified by LC/MS/MS. In addition, control samples were prepared along with the LPS-elicited samples under investigation in order to detect non-specific interactions which may have occurred between the PM proteins and the affinity matrices, i.e., without immobilization of LPS. Proteins that were identified in both the control and the LPS-immobilized samples were not considered, while those identified solely in MAMP-immobilized samples were considered as significant candidate LPS-interacting proteins.

#### 4.4.1. Detoxi-Gel™ Endotoxin Removing Gel Affinity Chromatography

The Detoxi-Gel™ Endotoxin Removing gel (Thermo Scientific, Rockford, IL, USA) uses a polymyxin B-based LPS-immobilization and was used as an affinity ligand in order to capture and enrich candidate LPS-interacting proteins. The agarose resin is bound to the polymyxin B ligand, which serves to attract and bind specifically to the lipid A moiety of the MAMPs used in this study, namely the LPS chemotypes from *Xcc* 8004 and 8530, thus allowing the O-chain polysaccharide to act as the bait. The affinity procedure followed was a method previously performed by Vilakazi et al. [9], but with modification of the manufacturer’s instructions to ensure high protein yields from the chromatographic technique.

#### 4.4.2. EndoTrap^®^ HD Endotoxin Removal Affinity Chromatography

The ligand utilized by the EndoTrap^®^ HD Endotoxin (Hyglos, Bernried, Germany) system is a bacteriophage-derived protein that has affinity for LPS from Gram-negative bacteria (in this case, the LPS chemotypes from *Xcc* 8004 and 8530) and specifically binds to the inner core, hence resulting in the lipid A and O-chain polysaccharide to act as bait for candidate LPS-interacting PM-associated proteins [9].

#### 4.4.3. MagReSyn™ Streptavidin Magnetic Polymeric Microsphere Affinity Chromatography

This procedure depends on derivatization of LPS (*Xcc* 8004 and 8530) and immobilization of the biotinylated lipid A and O-chain moieties, thus leaving the core oligosaccharide as the moiety for capture. The affinity chromatography technique, using MagReSyn™ streptavidin beads (ReSyn Biosciences, AEC-Amersham, Midrand, South Africa), followed a modification of the manufacturer’s instructions and an optimized technique of Vilakazi et al. [9]. A similar approach was followed by Giangrande et al. [23].

Finally, for all the affinity strategies, the enriched protein samples were pooled and concentrated to approximately 1.25 mg/mL prior to analysis by liquid chromatography-mass spectrometry tandem mass spectrometry (LC/MS/MS). In this regard, fractions were precipitated with 80% (*v*/*v*) analytical grade acetone, with four parts of ice-cold acetone added to one part of fraction, and incubation at −20 °C overnight. Thereafter, the tubes were centrifuged at 13,000× *g* for 10 min at RT, where after, the supernatants were discarded and the pellets subjected to three washes with ice-cold 80% (*v*/*v*) acetone, which included vortexing, re-suspending the pellets, and centrifuging at 13,000× *g* for 10 min per wash. After the washes, the acetone was discarded and the pellets were allowed to air dry and were resuspended in 2% SDS and 50 mM ammonium bicarbonate.

### 4.5. Preparation of Samples for Protein Identification by Liquid Chromatography-Mass Spectrometry (LC/MS)

#### 4.5.1. In-Solution Sample Preparation: Affinity Chromatography-Enriched Samples

In preparation for the on-bead hydrophilic interaction liquid chromatography (HILIC) magnetic bead workflow, the supplier’s beads were aliquoted into a new tube and the shipping solution was removed. Beads were then washed with 250 μL wash buffer [15% (*v*/*v*) acetonitrile (ACN), containing 100 mM ammonium acetate, pH 4.5] for 1 min. The beads were re-suspended in loading buffer containing [30% (*v*/*v*) ACN, 200 mM ammonium acetate, pH 4.5]. A total of 50 μg of protein was transferred to a 96-well protein LoBind plate (Merck, Darmstadt, Germany). Protein was reduced with tris(2-carboxylethyl)phosphine (TCEP) (Sigma, St. Louis, MO, USA), which was added to a final concentration of 10 mM and incubated at 60 °C for 1 h. Samples were cooled to RT and then alkylated with methylmethane-ethiosulphonate, MMTS (Sigma, St. Louis, MO, USA), which was added to a final concentration of 10 mM and incubated at RT for 15 min. HILIC magnetic beads were added at an equal volume to that of the sample and a ratio of 5:1 (*v*/*v*) total protein. The plate was then incubated at RT on the shaker at 900 rpm for 30 min to ensure binding of protein to the beads. After binding, the beads were washedtimes with 500 μL of 95% (*v*/*v*) ACN for 1 min. For digestion, 0.02 mg/mL trypsin (Promega, Madison, WI, USA) made up in 50 mM triethylammonium bicarbonate (TEAB) was added to the protein sample at a ratio of 1:10 (*v*/*v*) and the plate was incubated at 37 °C on the shaker for 4 h. After digestion, the supernatant containing peptides was removed and dried. Samples were then re-suspended in LC loading buffer: 0.1% (*v*/*v*) formic acid (FA) in 2.5% (*v*/*v*) ACN prior to analysis by LC/MS/MS.

#### 4.5.2. Liquid Chromatography-Mass Spectrometry (LC/MS) Analysis

Analysis of in-solution protein samples was conducted at the Centre for Proteomic and Genomic Research (CPGR, Cape Town, South Africa). LC/MS analysis utilized a Q-Exactive quadrupole-Orbitrap mass spectrometer (ThermoFisher Scientific, Waltham, MA, USA) coupled with a Dionex Ultimate 3000 nano-ultra performance liquid chromatography (UPLC) system. Data were acquired using: Xcalibur v4.1.31.9, Chromeleon v6.8 (SR13), Orbitrap MS v2.9 (build 2926), and Thermo Foundations 3.1 (SP4). Peptides were dissolved in 0.1% (*v*/*v*) FA, 2.5% (*v*/*v*) ACN and loaded on a C18 trap column (PepMap100, 300 μm × 5 mm × 5 μm). The solvent system employed was solvent A: LC water (Burdick and Jackson, Muskegon, MI, USA) containing 1% (*v*/*v*) FA and solvent B: ACN containing 0.1% (*v*/*v*) FA. Samples were trapped onto the column at 2% solvent B and washed for 3 min before the valve was switched and peptides were eluted onto the analytical column as described hereafter. Chromatographic separation was performed with a Waters nanoEase (Zenfit) M/Z Peptide CSH C18 column (75 μm × 25 cm × 1.7 μm) (Waters Corporation, Milford, MA, USA). The multi-step gradient for peptide separation was generated at 300 nL/min as follows: time change 5 min, gradient change: 2–5% Solvent B; time change 40 min, gradient change: 5–18% Solvent B; time change 10 min, gradient change: 18–30% Solvent B; time change 2 min, gradient change: 30–80% Solvent B. The gradient was then held at 80% solvent B for 10 min before returning to 2% solvent B for 5 min. To ensure carryover did not occur between runs, a wash step was included at the end of the run, which comprised a gradient change of 2–80% Solvent B at 35 min. The gradient was held at 80% Solvent B for 5 min before returning to 2% Solvent B and conditioning the column for 15 min. All data acquisition was performed using Proxeon stainless steel emitters (ThermoFisher, Waltham, MA, USA). The mass spectrometer was operated in a positive ionization mode with a capillary temperature of 320 °C. The applied electrospray voltage was 1.95 kV.

### 4.6. Data Analysis

The data interrogation was performed using the PMI-Byonic-com v2.6.46 Byonic Software (Protein Metrics, Cupertino, CA, USA). The *A. thaliana* reference proteome was sourced from Uniprot Knowledgebase (UniprotKB, www.uniprot.org) database whereby the spectra from peptide fragments resulting from MS/MS were matched. The fragments were obtained by collision induced dissociation (CID) low energy. The following search parameters were used: trypsin enzyme, cutting at the C-ends of lys and arg; the fixed modification was carbidomethyl (M); and the variable modifications were deamidated (NQ) and oxidation (Methionine). The maximum number of missed cleavages was 2. The precursor mass tolerance was 10 ppm and the fragment mass tolerance was 20 ppm. The protein false discovery rate (FDR) cut-off was 1% and the best score range was between 0–1000 where a score of greater than 300 is considered significant [60,61]. Once the mass spectra of the fragmented peptides were obtained, they were analyzed by the Byonic™ software and searched against the UniprotKB database, resulting in peptide spectrum matches (PSMs). The PSMs consider various criteria for protein identification such as the number of unique peptides (the total number of PSMs for the protein, excluding the duplicates). The PSMs were ranked for significance and confident identifications according to two plots, namely the score plot and the mass error loadings plot (Figure S7). The score plot indicates the variation that exists between the two groups of data (forward and reverse proteins) separated by the score ranking, resulting in the variable selection and the differentially abundant proteins. The variable importance in projection (VIP) method is responsible for the variable selection whereby the proteins are ranked based on contribution to the total variation. This sets a threshold for selecting variable and differentially abundant proteins with a VIP score greater than 1 [62]. The value of 1 adds greatly to the significance of the protein as it represents the log probability thereof, which is one of two parameters that guide significant and confident protein identifications. The second is the Byonic score significance according to the Byonic™ software. The two parameters are used in conjunction to determine the confident identifications of the differential proteins in order to greatly increase the significance. The obtained dataset was then compared to the peptides of the UniprotKb database to identify the *A. thaliana* PM-associated LPS-interacting candidate proteins.

## 5. Conclusions

Bacterial MAMPs such as LPS have been reported as inducers of MTI and subsequent defense-related responses following recognition by the PRRs of a host plant [63]. While RLKs have been implicated in the said perception [9,11,19], the exact LPS binding mechanism(s) and recognition as well as the subsequent signaling cascades are poorly understood. In addition, it has been speculated that different moieties within the tripartite lipoglycan may bind to different PRRs [8]. In this regard, the current study investigated proteomic approaches to enable the identification of candidate LPS-interacting proteins from *A. thaliana* PM fractions following treatment with two LPS chemotypes from both the *Xcc* wild-type 8004, having the typical moieties for a “smooth”-type LPS and the *Xcc* mutant 8530 strain, which has a modified lipid A, truncated core oligosaccharide, and the absence of an O-chain. The identified proteins belonged to three functional categories mainly related to this investigation, namely perception and signaling, defense and stress response, and membrane transport and trafficking (additional proteins belonging to skeletal structure and metabolic processes were also identified). In particular, those pertaining to the first two categories are thought to be important in the initial response to both LPS chemotypes, even though the mutant strain exhibited altered structural moieties with different features. However, the majority of the significant proteins pertaining to perception and signaling were identified in response to the wild-type LPS. Furthermore, the EndoTrap^®^ HD affinity strategy was considerably affected by the molecular differences of the mutant LPS moieties (where the inner core is bound to the matrix, thus resulting in the lipid A and O-chain as bait) and hence, this influenced the enrichment of candidate MAMP-interacting proteins. In addition, the majority of common/shared candidate LPS-interacting PM-associated proteins were identified between the polymyxin B-immobilized and MagReSyn™ affinity strategies, which increased the significance of identification. Common candidate LPS-interacting PM-associated proteins were also identified between the two LPS chemotypes. This also suggests that these proteins may assemble in a concerted/coordinated manner in a “complex” when responding to the LPS as seen in flg22 perception, where the FLS2 receptor forms a complex with BAK1 and BIK1 to activate MTI signaling [33]. Similar assemblies of proteins have been implicated in LPS perception [9,19]. A significant common candidate LPS-interacting PM-associated protein from the current study is the putative BPI/LBP family protein At1g04970, identified at 18 and 24 h in response to the *Xcc* 8004 LPS treatment, as well as at 6, 12, and 24 h following *Xcc* 8530 LPS treatment, which was also identified in two enrichment strategies. Iizasa et al. [13] demonstrated for the first time that the N-terminal domain of the AtLBP/BPI-related1 (AtLBR1) in *A. thaliana* binds directly to LPS.

Proteins identified in individual affinity enrichments highlighted the importance of distinct LPS moieties e.g., lectin RLKs only in response to wild-type LPS. SD-RLK At1g11330/ SD1-13 enriched at an early 6 h time point post-LPS treatment is a close homolog of the *Nt-Sd-RLK* found to specifically be upregulated in response to LPS in tobacco at both early and late responses [11]. Thus, it is plausible to consider that S-domain RLKs and in particular, the SD-RLK 1-13, may be involved in the proposed receptor complex in response to the O-chain polysaccharide in wild-type LPS in *A. thaliana*, thus suggesting that the moieties of LPS influences its perception by the plant. In addition, a G-type LecRLK, At1g67520, was enriched 6 h post-wild-type LPS treatment. The encoding gene of this protein has been shown to be upregulated in response to LPS [32].

The inactive BIR2 and SOBIR1 were also identified in this study, enriched in the 6 and 24 h treated fractions following *Xcc* 8004 LPS elicitation. These are most likely regulators of MTI in response to LPS and have been reported to play significant roles in BAK1-BRI1 signalling. Vilakazi et al. [9] and Baloyi et al. [19] have previously identified BAK1 in playing a role in perceiving LPS.

In conclusion, the affinity strategies that were investigated in this study have enabled the identification of candidate PM-associated proteins that possibly interact with and likely respond to the LPS moieties. In so doing, an interesting perspective of the probable perception and signaling in terms of an LPS receptor/receptor complex and subsequent related plant defense responses was gained.

## Figures and Tables

**Table 1:** Commonly-enriched candidate LPS-interacting PM-associated proteins from the three affinity chromatography systems with LPS chemotype *Xcc* 8004 as ligand (compiled from Tables S1, S4 and S7).

Protein *^a^*	Accession Number *^b^*
**Common proteins bound to LPS *Xcc* 8004 -functionalized Polymyxin B and MagReSyn^TM^**	
Putative GTP-binding protein ara-3	Q9FJF1
Putative permeability-increasing protein (BPI)/LPS-binding protein (LBP) family protein At1g04970	Q9MAU5
MLP-like protein 423	Q93VR4
Heat shock protein 70 (Hsp 70) family protein	F4K007
Ras-related protein RABE1c	P28186
Ras-related protein RABA1f	Q9FJH0
Ras-related protein RABG3a	Q948K8
V-type proton ATPase subunit G1	O82628
V-type proton ATPase subunit d2	Q9LHA4
Tubulin alpha-5 chain	B9DHQ0
Annexin D2	Q9XEE2
Tubulin beta-2 chain	Q56YW9
Nitrilase 1	P32961
Adenine phosphoribosyl transferase 1	F4HSX1
**Common proteins bound to LPS *Xcc* 8004-functionalized MagReSyn^TM^ and EndoTrap^®^**	
Mannose-binding lectin superfamily protein	A0A1I9LQM9
ATPase 2	P19456
Plasma membrane ATPase	F4JPJ7
Aldolase-type TIM barrel family protein	A8MS37

***^a^*** the protein identified by LC/MS/MS. ***^b^*** the accession number of the proteins. The perception and signaling proteins are highlighted in red, the defense and response proteins are highlighted in blue, and the membrane trafficking and transport are highlighted in green. The proteins highlighted in black pertain to structure and metabolic process.

**Table 2:** Commonly-enriched candidate LPS-interacting PM-associated proteins from the three affinity chromatography systems with LPS chemotype *Xcc* 8530 as ligand. The table descriptions are as for those in Table 1 and were compiled from Tables S2, S5 and S8.

Protein *^a^*	Accession Number *^b^*
**Common proteins bound to LPS *Xcc* 8530-functionalized Polymyxin B and MagReSyn™**	
Putative MO25-like protein At4g17270	Q9M0M4
Membrane steroid-binding protein 2	Q9M2Z4
Putative BPI/LBP family protein At1g04970	Q9MAU5
GPI-anchored adhesin-like protein	Q9FF91
Probable inactive receptor kinase At3g02880	Q9M8T0
Probable LRR-RLK ser/thr-protein kinase At3g14840	C0LGN2
UPF0496 protein At3g28310/At3g28320	Q9M386
Phosphatidylinositol 4-kinase alpha 1	Q9SXA1
B-cell receptor-associated 31-like protein	Q93XZ7
Jacalin-related lectin 5	Q9ZU23
Heat shock protein 70 (Hsp 70) family protein	F4K007
Cytochrome P450 83B1	O65782
Cysteine-rich RLK (Receptor-like protein kinase) 10	A0A1P8B597
Aluminum induced protein with YGL and LRDR motifs	Q9FG81
Remorin	O80837
Dehydrin ERD14	P42763
MLP-like protein 423	Q93VR4
Receptor-like protein 51	Q9SN38
Remorin family protein	F4KEA0
Hypersensitive-induced response protein 4	Q9FHM7
Ras-related protein RABG3a	Q948K8
Sugar transporter ERD6-like 4	Q93YP9
Nuclear transport factor 2 (NTF2) family protein	Q9FMC7
Plasma-membrane associated cation-binding protein 1	F4JUT9
V-type proton ATPase subunit G1	O82628
Patellin-3	Q56Z59
ATPase 11	Q9LV11
Calcium-transporting ATPase 10	Q9SZR1
ABC-2 type transporter family protein	A0A1P8BAZ0
Ammonium transporter 1-like protein	Q93Z11
Sodium/calcium exchanger NCL	Q8L636
Syntaxin-132	Q8VZU2
MD-2-related lipid recognition domain-containing protein	Q9SF20
Annexin D2	Q9XEE2
Tubulin beta-2 chain	Q56YW9
Tubulin alpha-5 chain	B9DHQ0
Actin 2	F4J8V9
Nitrilase 1	P32961
Adenine nucleotide alpha hydrolases-like superfamily protein	Q9M328
Cytochrome P450 71B28	Q9SAE3
Probable fructokinase-1	Q9SID0
Phosphoglucomutase/phosphomannomutase family protein	F4I6W4
**Common proteins bound to LPS *Xcc* 8530 functionalized MagReSyn^TM^ and EndoTrap^®^**	
Aldolase-type TIM barrel family protein	A8MS37

***^a^*** the protein identified by LC/MS/MS. ***^b^*** the accession number of the proteins. The perception and signaling proteins are highlighted in red, the defense and response proteins are highlighted in blue, and the membrane trafficking and transport are highlighted in green. The proteins highlighted in black pertain to structure and metabolic process.

**Table 3:** Commonly-enriched candidate LPS-interacting PM-associated proteins for both LPS chemotypes subsequent to the polymyxin B affinity chromatography strategy. The table descriptions are as for those in Table 1 and are compiled from Tables S1 and S2.

Protein *^a^*	Accession Number *^b^*
Carbohydrate-binding-like fold	Q9LZQ4
General regulatory factor 10	F4I1C1
Probable LRR receptor-like ser/thr-protein kinase At1g53430	C0LGG8
Probable LRR receptor-like ser/thr-protein kinase At3g14840	C0LGN2
Phototropin-1	O48963
L-type lectin-domain containing receptor kinase IV.1	O80939
Phospholipase D alpha 1	Q38882
Calcium-dependent protein kinase 3	Q42479
Putative MO25-like protein At4g17270	Q9M0M4
Probable inactive receptor kinase At3g02880	Q9M8T0
Phosphatidylinositol 4-kinase alpha 1	Q9SXA1
Calcium-dependent protein kinase 21	Q9ZSA2
LRR transmembrane protein kinase	F4HRH4
Nucleotide-diphospho-sugar transferases superfamily protein	F4IVY1
Receptor like protein 54	F4KHA2
Low-density receptor-like protein	Q8H0X5
B-cell receptor-associated 31-like protein	Q93XZ7
GPI-anchored adhesin-like protein	Q9FF91
Phospholipase D	F4JNU6
Membrane steroid-binding protein 2	Q9M2Z4
Putative BPI/LBP family protein At1g04970	Q9MAU5
GF14 protein phi chain	F4HWQ5
Cysteine-rich RLK (Receptor-like protein kinase) 10	A0A1P8B597
Calcium-dependent protein kinase 15	F4JKC7
Late embryogenesis abundant protein, group 2	O80576
Cytochrome P450 83B1	O65782
Remorin	O80837
Dehydrin ERD14	P42763
Protein BONZAI 2	Q5S1W2
MLP-like protein 423	Q93VR4
Hypersensitive-induced response protein 4	Q9FHM7
Callose synthase 12	Q9ZT82
Jacalin-related lectin 5	Q9ZU23
Aluminum induced protein with YGL and LRDR motifs	F4JZM6
Remorin family protein	F4KEA0
Late embryogenesis abundant (LEA) hydroxyproline-rich glycoprotein family	Q9M386
Heat shock protein 70 (Hsp 70) family protein	F4K007
Ras-related protein RABE1c	P28186
Ras-related protein RABG3a	Q948K8
Synaptotagmin A	F4IFM6
Oligopeptide transporter 3	O23482
V-type proton ATPase subunit G1	O82628
Aquaporin PIP2-2	P43287
Chloride channel protein CLC-a	P92941
Aquaporin PIP2-7	P93004
Patellin-3	Q56Z59
Sodium/calcium exchanger NCL	Q8L636
ABC transporter B family member 2	Q8LPK2
Potassium transporter 13	Q8LPL8
Putative ion channel POLLUX-like 1	Q8VZM7
Syntaxin-132	Q8VZU2
Sugar transporter ERD6-like 4	Q93YP9
Potassium transporter 7	Q9FY75
Calcium-transporting ATPase 8	Q9LF79
Novel plant SNARE 13	Q9LRP1
ATPase 11	Q9LV11
Alpha-soluble NSF attachment protein 2	Q9SPE6
Calcium-transporting ATPase 10	Q9SZR1
Probable aquaporin PIP2-6	Q9ZV07
Putative plant snare 13	F4J563
Major facilitator superfamily protein	Q9FMT8
Pyrophosphate-energized vacuolar membrane proton pump 1	P31414
Aquaporin PIP1-2	Q06611
ABC transporter G family member 15	Q8RWI9
Patellin-6	Q9SCU1
SecY protein transport family protein	Q8RWJ5
Ammonium transporter 1-like protein	Q93Z11
Tubulin alpha-5 chain	B9DHQ0
Actin-7	P53492
Annexin D2	Q9XEE2
Tubulin beta-2 chain	Q56YW9
Probable elongation factor 1-gamma 1	O04487
Actin-8	Q96293
Actin 2	F4J8V9
Nitrilase 1	P32961
Cytochrome P450 72A15	Q9LUC5
Cytochrome P450 71B28	Q9SAE3
Cytochrome P450 71A22	Q9STL1
Triacylglycerol lipase-like 1	F4HRB4
Adenine nucleotide alpha hydrolases-like superfamily protein	Q94II5
Formate-tetrahydrofolate ligase	Q9SPK5
Adenine phosphoribosyl transferase 1	F4HSX1

***^a^*** the protein identified by LC/MS/MS. ***^b^*** the accession number of the proteins. The perception and signaling proteins are highlighted in red, the defense and response proteins are highlighted in blue, and the membrane trafficking and transport are highlighted in green. The proteins highlighted in black pertain to structure and metabolic process.

**Table 4:** Commonly-enriched candidate LPS-interacting PM-associated proteins for both the LPS chemotypes subsequent to the EndoTrap^®^ HD Endotoxin removal affinity chromatography strategy. The table descriptions are as for those in Table 1 and compiled from Tables S4 and S5.

Protein	Accession Number
Myrosinase-binding protein 1	Q9SAV0
Annexin D1	Q9SYT0
Aldolase-type TIM barrel family protein	A8MS37

The defense and response proteins are highlighted in blue.

**Table 5:** Commonly-enriched candidate LPS-interacting PM-associated proteins for both the LPS chemotypes subsequent to the MagReSyn™ streptavidin magnetic polymeric microsphere affinity chromatography strategy. The table descriptions are as for those in Table 1 and are compiled from Tables S7 and S8.

Protein *^a^*	Accession Number *^b^*
Ser/thr-protein kinase BSK7	F4I3M3
Plasma-membrane associated cation-binding protein 1	F4JUT9
Putative BPI/LBP family protein At1g04970	Q9MAU5
Hypersensitive-induced response protein 3	Q9SRH6
Heat shock protein 70 (Hsp 70) family protein	F4K007
MLP-like protein 423	Q93VR4
Aluminium induced protein with YGL and LRDR motifs	Q9FG81
Ras-related protein RABA1f	Q9FJH0
Ras-related protein RABA5c	P28187
Ras-related protein RABG3a	Q948K8
Ras-related protein RABF2b	Q9SN68
Ras-related protein RABD2c	Q9SEH3
Ras-related protein RABB1c	P92963
V-type proton ATPase subunit G1	O82628
ATPase 2	P19456
V-type proton ATPase subunit d2	Q9LHA4
Plasma membrane ATPase	F4JPJ7
ADP-ribosylation factor A1F	Q6ID97
Tubulin beta-2 chain	Q56YW9
Tubulin alpha-5 chain	B9DHQ0
Annexin D2	Q9XEE2
Aldolase-type TIM barrel family protein	A8MS37
Nitrilase 1	P32961
Temperature-induced lipocalin-1	Q9FGT8

***^a^*** the protein identified by LC/MS/MS. ***^b^*** the accession number of the proteins. The perception and signaling proteins are highlighted in red, the defense and response proteins are highlighted in blue, and the membrane trafficking and transport are highlighted in green. The proteins highlighted in black pertain to structure and metabolic process.

## Supplementary Materials

The following are available online at https://www.mdpi.com/2076-0817/9/10/787/s1, Figure S1: A representative 12% SDS-PAGE gel subsequent to sucrose-density gradient isolation showing the three isolated fractions., Figure S2: A representative Western blot analysis., Figure S3: The elution profile of the binding and desorption events between the LPS-immobilized polymyxin B and interacting PM-associated candidate proteins for the 6 h time study from the *A. thaliana.*, Figure S4: The elution profile of the binding and desorption events between the LPS-immobilized to the bacteriophage EndoTrap^®^ resin and interacting PM-associated candidate proteins for the 6 h time study from *A. thaliana*., Figure S5: The elution profiles of the binding and desorption events between the LPS-immobilized MagReSyn™ streptavidin polymeric microspheres and interacting PM-associated candidate proteins from *A. thaliana* 6 h treatment., Figure S6: A 12.5% SDS-PAGE gel of LPS from *B. cepacia*, *Xcc* 8004 and *Xcc* 8530, as well as gel of the biotinylated *Xcc* 8004 and *Xcc* 8530 LPS chemotypes., Figure S7: Representation of the data obtained by the Byonic™ software for protein identification. Table S1: The candidate LPS-interacting PM associated proteins identified by LC/MS/MS after enrichment with LPS-immobilized polymyxin B affinity chromatography for the time study 0, 6, 12, 18 and 24 h, subsequent to *A. thaliana* treatment with the *Xcc* 8004 LPS chemotype., Table S2: The candidate LPS-interacting PM-associated proteins identified by LC/MS/MS after enrichment with LPS-immobilized polymyxin B affinity chromatography for the time study 0, 6, 12, 18 and 24 h subsequent to *A. thaliana* treatment with the *Xcc* 8530 LPS chemotype., Table S3: PM proteins that interacted with the polymyxin B resin (no LPS immobilized) used for affinity chromatography and identified by LC/MS/MS., Table S4: The candidate LPS-interacting PM-associated proteins identified by LC/MS/MS after enrichment with the EndoTrap^®^ HD affinity chromatography for the time study 0, 6, 12, 18 and 24 h subsequent to *A. thaliana* treatment with LPS chemotype *Xcc* 8004., Table S5: The candidate LPS-interacting PM-associated proteins identified by LC/MS/MS after the enrichment with the EndoTrap^®^ HD affinity chromatography for the time study 0, 6, 12, 18 and 24 h subsequent to *A. thaliana* treatment with LPS chemotype *Xcc* 8530., Table S6: The PM proteins that interacted with the EndoTrap^®^ HD affinity chromatography resin (no LPS immobilized) and identified by LC/MS/MS., Table S7: The candidate LPS-interacting PM-associated proteins identified by LC/MS/MS after the enrichment with the LPS-immobilized MagReSyn™ streptavidin polymeric microspheres affinity chromatography for the time study 0, 6, 12, 18 and 24 h subsequent to *A. thaliana* treatment with LPS chemotype *Xcc* 8004., Table S8: The candidate LPS-interacting PM-associated proteins identified by LC/MS/MS after the enrichment with the LPS-immobilized MagReSyn™ streptavidin polymeric microspheres affinity chromatography for the time study 0, 6, 12, 18 and 24 h subsequent to *A. thaliana* treatment with LPS chemotype *Xcc* 8530., Table S9: The PM proteins that interacted with the MagReSyn™ streptavidin polymeric microspheres (no LPS immobilized) and identified by LC/MS/MS., Supplementary Data Files: Listed according to functional categories.
