# Supplementary material for: Identification of MAMP-Responsive Plasma Membrane-Associated Proteins in Arabidopsis thaliana Following Challenge with Different LPS Chemotypes from Xanthomonas campestris
Source: Pathogens. 2020 Sep 25;9(10):787. doi: 10.3390/pathogens9100787 (PMC7650673; doi:10.3390/pathogens9100787)
Supplement: Supplementary file 1 [file pathogens-09-00787-s001.zip › Supplementary Figures_Hussan et al_2020.docx]

Identification of MAMP-responsive plasma membrane-associated proteins in *Arabidopsis thaliana* following challenge with different LPS chemotypes from *Xanthomonas campestris*

Raeesa H. Hussan, Ian A. Dubery and Lizelle A. Piater*

Department of Biochemistry, University of Johannesburg, Auckland Park, 2006, South Africa;

raeesahussan@gmail.com (R.H.); idubery@uj.ac.za (I.D.)

***** Correspondence: lpiater@uj.ac.za (L.P.); Tel.: +27-11-559-2403

Received: date; Accepted: date; Published: date

**SUPPLEMENTARY FIGURES**

**HM**

250

150

100

70

50

40

20

15


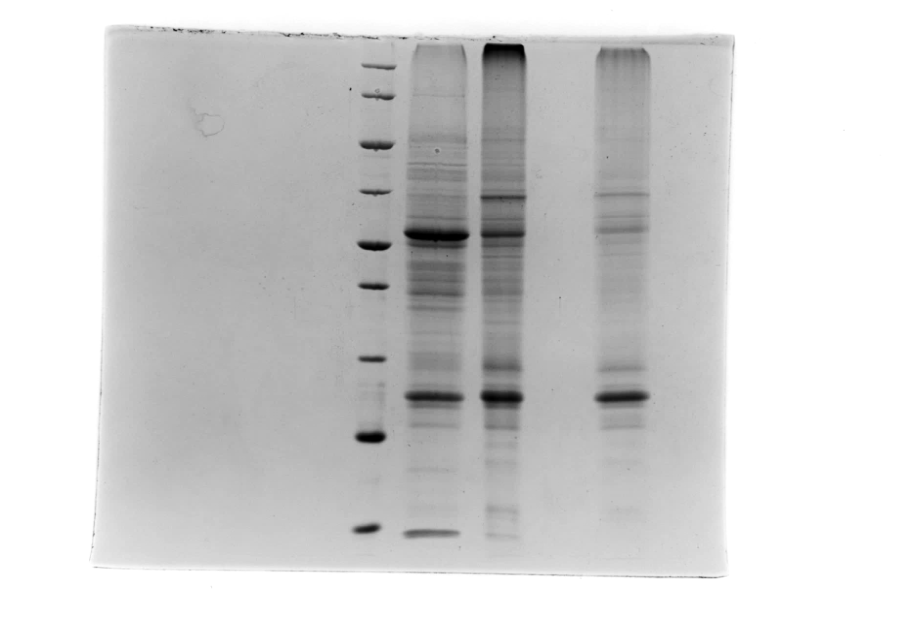


kDa

**MW**

**MF**

**PM**

**Figure S1**: A representative 12% SDS-PAGE gel subsequent to sucrose-density gradient isolation showing the three isolated fractions; lane 1: the Broad Range protein marker (ThermoFisher) lane 2: homogenate (HM) fraction, lane 3: microsomal fraction (MF) and lane 4: plasma membrane (PM) fraction. Subsequent to protein quantification 20 µg of each of the fractions was loaded onto the gel followed by electrophoresis at 90 V and Fairbanks staining.

Kda

**B)**

**A)**


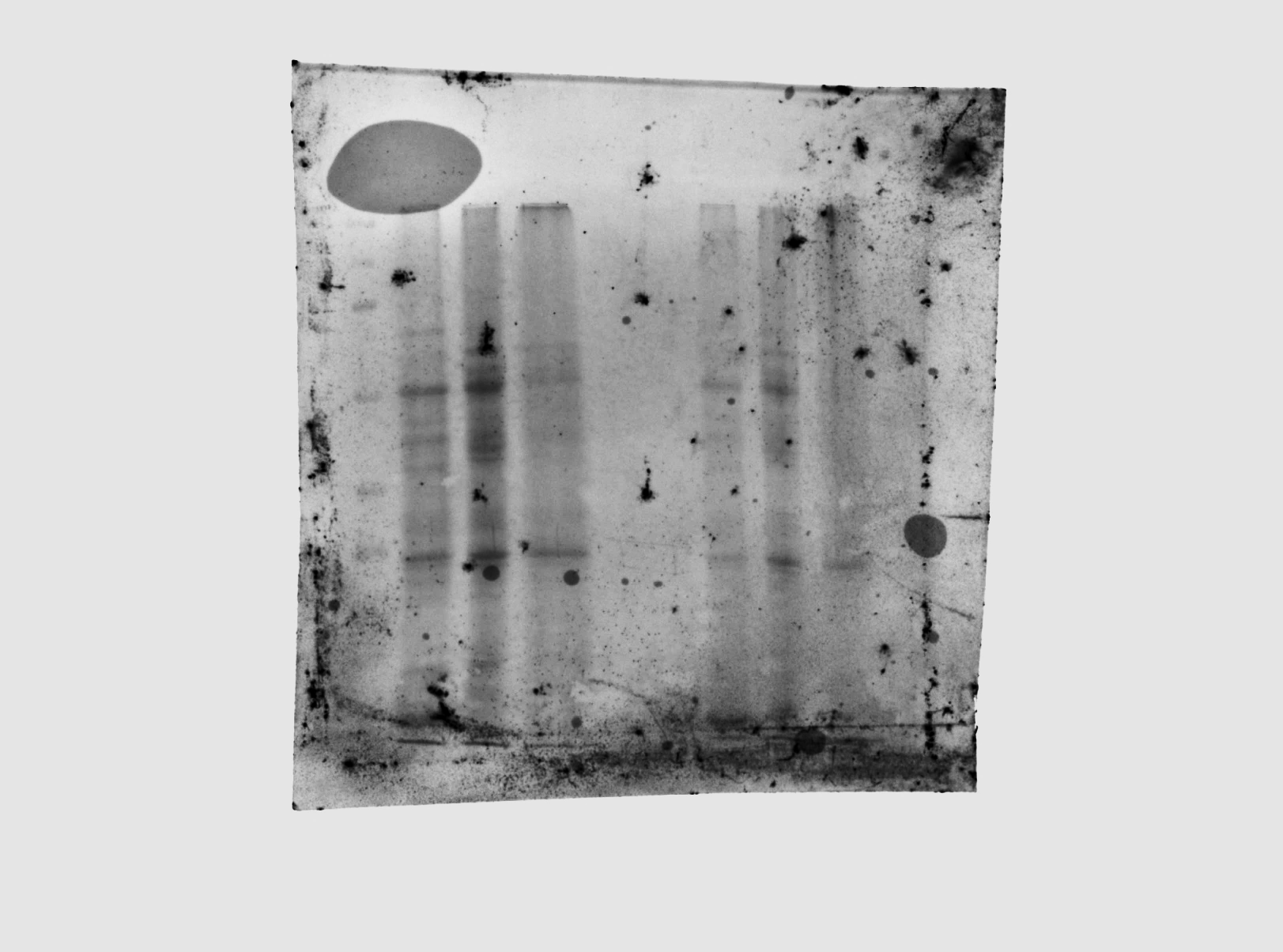

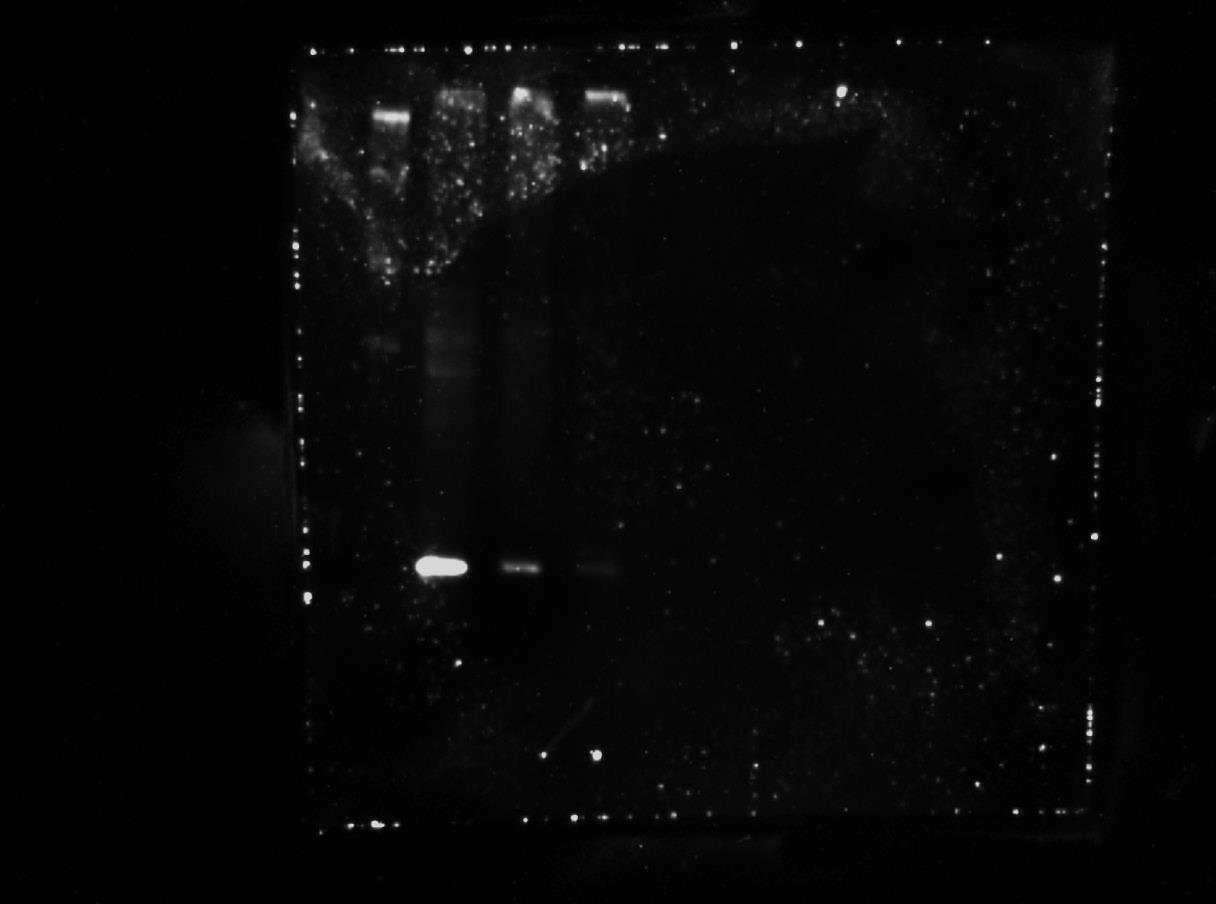


**HM MF PM**

**Figure S2**: A representative Western blot analysis with A) showing the presence of the MAPK detected in the 45 kDa range for the various fractions namely homogenate (HM), microsomal fraction (MF) and plasma membrane (PM) using anti-active MAPK antibody pAB, Rabbit, (pTEpY) (Promega), and B) an Amido Black stained nitrocellulose membrane serving as a loading control. Equal concentrations of the three fractions were loaded to show that the lack of the MAPK activity present in each fraction was not due to the absence/unequal loading of proteins.

**B)**

**A)**

**C)**

Figure **S3**: The elution profile of the binding and desorption events between the LPS-immobilized polymyxin B and interacting PM-associated candidate proteins from the *A. thaliana* for A) LPS chemotype *Xcc* 8004 and B) LPS chemotype *Xcc* 8530 following 6 h treatment. The blue curve represents the absorbance of the flow-through (non-bound proteins) fractions eluted with 10 mM Tris-HCl, the red curve represents the absorbance of non-specific bound proteins eluted with 1 M NaCl and the green curve indicates the absorbance of the candidate LPS-interacting PM-associated proteins desorbed from the column by 1% SDS eluent. **C**) Represents polymyxin B resin (no LPS immobilization) interaction with proteins for the non-specific binding control and were not considered to be interacting proteins.

**B)**

**A)**

**C)**

**Figure S4**: The elution profile of the binding and desorption events between the LPS-immobilized to the bacteriophage EndoTrap® resin and interacting PM-associated candidate proteins for the 6 h time study from *A. thaliana* for A) LPS chemotype treated *Xcc* 8004 and B) LPS chemotype treated *Xcc* 8530. The orange curve represents the absorbance of the flow-through (non-bound proteins) fractions eluted with 10 mM Tris-HCl, and the blue curve represents the absorbance of the PM-associated LPS-interacting candidate proteins desorbed from the column with the EndoTrap® HD equilibration buffer (EQ). **C**) Represents EndoTrap® resin (no LPS immobilization) interaction with proteins for the non-specific binding control and were not considered to be interacting protein.

**B)**

**A)**

**C)**

**Figure S5**: The elution profiles of the binding and desorption events between the LPS-immobilized MagReSyn™ streptavidin polymeric microspheres and interacting PM-associated candidate proteins from *A. thaliana* 6 h treatment of A) LPS chemotype *Xcc* 8004 and B) LPS chemotype *Xcc* 8530. The blue curve represents the absorbance of the flow through (non-bound proteins) fraction eluted with 10 mM Tris-HCl from the resin. The orange curve represents the absorbance of the non-specific fraction of proteins that are eluted with 1 M NaCl and lastly the black curve represents the candidate LPS-interacting PM-associated proteins that are eluted with 1% SDS. **C**) Represents MagReSyn™ streptavidin polymeric microsphere (no LPS immobilization) interaction with proteins for the non-specific binding control and were not considered to be interacting protein.

**B)**

O-chain

***Xcc* 8004**

***Xcc* 8530**

Lipid A


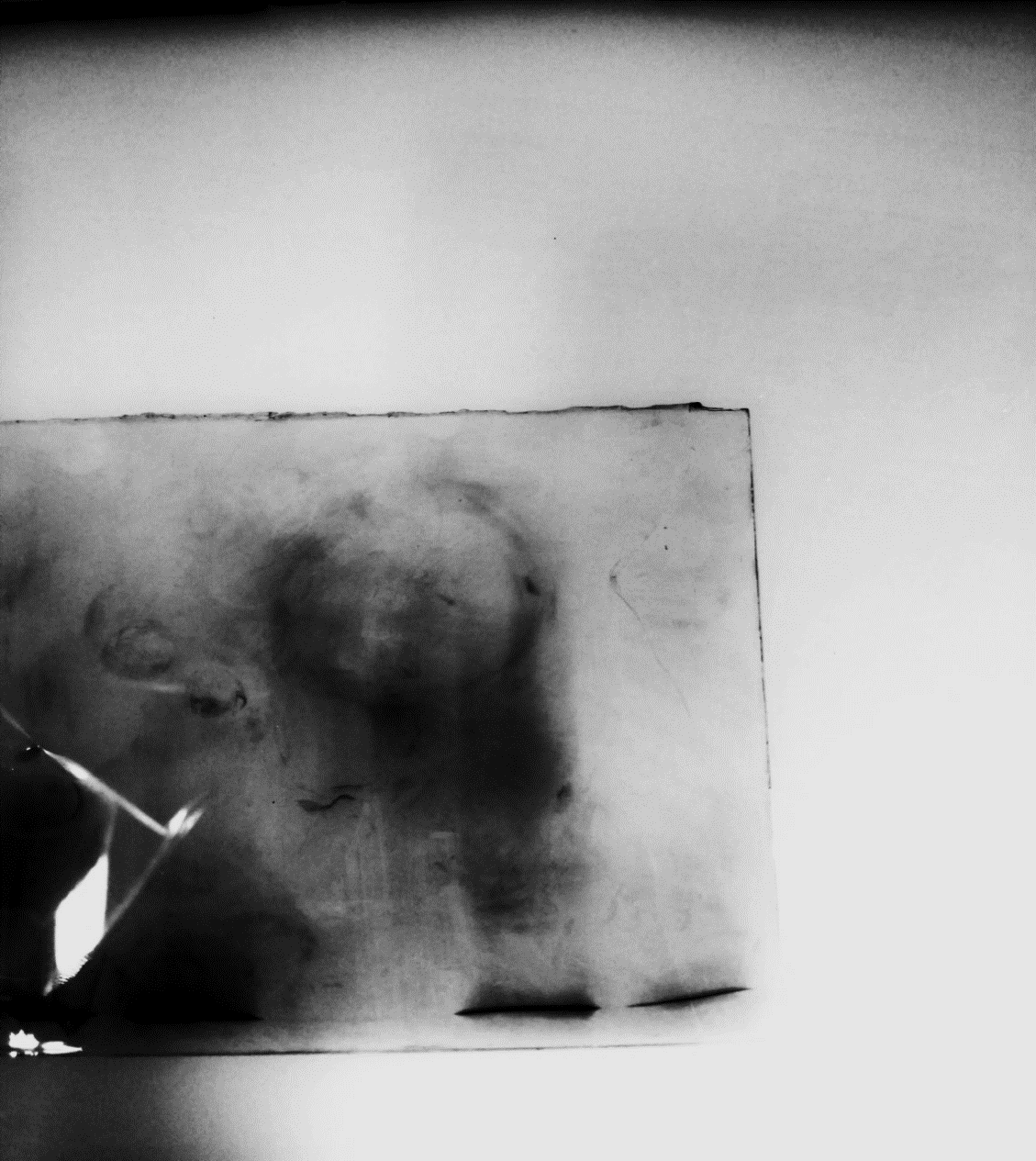


Lipid A

***B. cep***

***Xcc* 8004**

***Xcc* 8530**

Core oligosaccharide


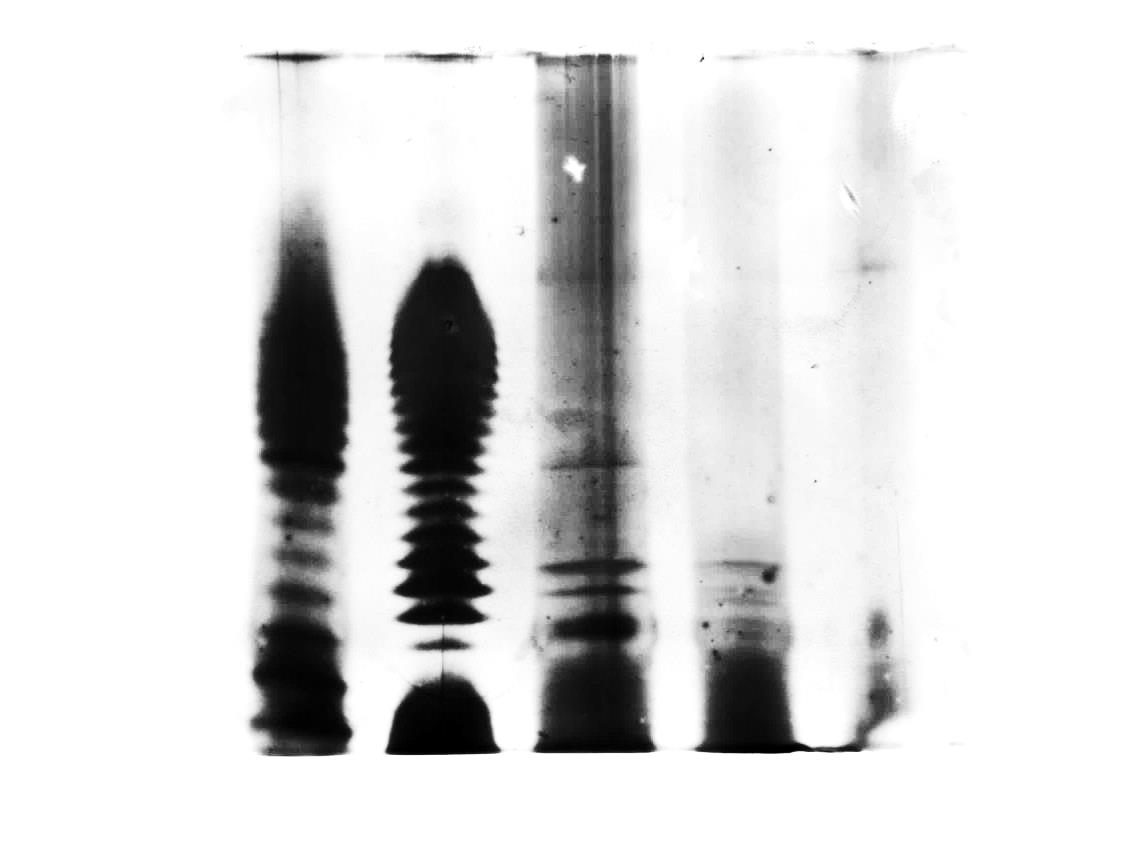


O-chain


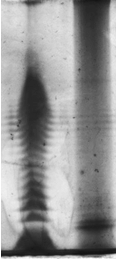


**A)**

**Figure S6**: **A**) A 12.5% SDS-PAGE gel of LPS from *B. cepacia*, *Xcc* 8004 and *Xcc* 8530. Stock solutions of 1 mg/mL each of the LPS were prepared by diluting 1:1 (v/v) with sample buffer, and 30 µL was loaded and electrophoresed. The LPS banding patterns obtained were visualized by silver-periodate staining whereby the carbohydrates were oxidized by periodic acid. The components of LPS are indicated on the gel. **B**) A 12.5% SDS-PAGE gel of the biotinylated *Xcc* 8004 and *Xcc* 8530 LPS chemotypes. Two mg/mL of the LPS samples were prepared, electrophoresed at 12-15 mA and subsequently silver stained.


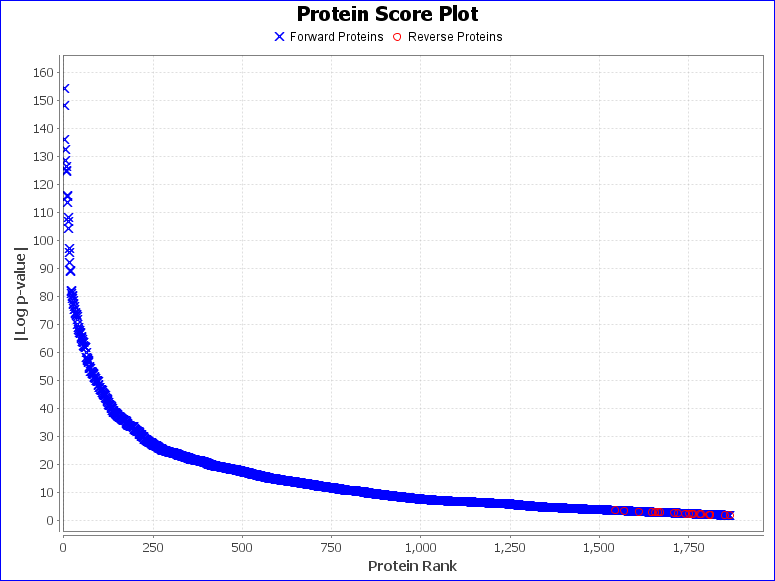

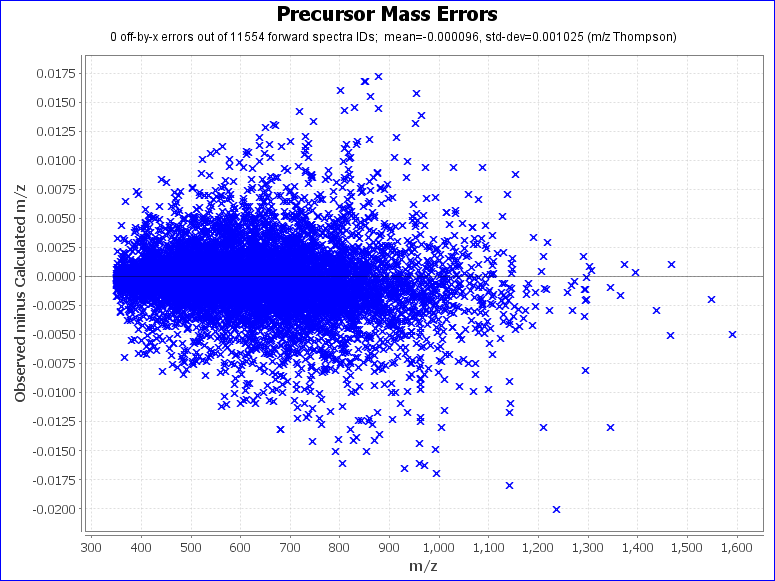


**B)**

**A)**

**Figure S7**: Representation of the data obtained by the Byonic™ software for protein identification. **A**) A score plot indicating the differential abundance of proteins and **B**) the computed mass which is observed in the precursor mass error loadings plot *i.e*. the difference between the calculated mass and the observed mass of the peptides.
